# survex: an R package for explaining machine learning survival models

**DOI:** 10.1093/bioinformatics/btad723

**Published:** 2023-12-01

**Authors:** Mikołaj Spytek, Mateusz Krzyziński, Sophie Hanna Langbein, Hubert Baniecki, Marvin N Wright, Przemysław Biecek

**Affiliations:** MI2.AI, Faculty of Mathematics and Information Science, Warsaw University of Technology, Warsaw, Poland; MI2.AI, Faculty of Mathematics and Information Science, Warsaw University of Technology, Warsaw, Poland; Leibniz Institute for Prevention Research and Epidemiology—BIPS, Bremen, Germany; Faculty of Mathematics and Computer Science, University of Bremen, Bremen, Germany; MI2.AI, Faculty of Mathematics and Information Science, Warsaw University of Technology, Warsaw, Poland; MI2.AI, Faculty of Mathematics, Informatics and Mechanics, University of Warsaw, Warsaw, Poland; Leibniz Institute for Prevention Research and Epidemiology—BIPS, Bremen, Germany; Faculty of Mathematics and Computer Science, University of Bremen, Bremen, Germany; Section of Biostatistics, Department of Public Health, University of Copenhagen, Copenhagen, Denmark; MI2.AI, Faculty of Mathematics and Information Science, Warsaw University of Technology, Warsaw, Poland; MI2.AI, Faculty of Mathematics, Informatics and Mechanics, University of Warsaw, Warsaw, Poland

## Abstract

**Summary:**

Due to their flexibility and superior performance, machine learning models frequently complement and outperform traditional statistical survival models. However, their widespread adoption is hindered by a lack of user-friendly tools to explain their internal operations and prediction rationales. To tackle this issue, we introduce the survex R package, which provides a cohesive framework for explaining any survival model by applying explainable artificial intelligence techniques. The capabilities of the proposed software encompass understanding and diagnosing survival models, which can lead to their improvement. By revealing insights into the decision-making process, such as variable effects and importances, survex enables the assessment of model reliability and the detection of biases. Thus, transparency and responsibility may be promoted in sensitive areas, such as biomedical research and healthcare applications.

**Availability and implementation:**

survex is available under the GPL3 public license at https://github.com/modeloriented/survex and on CRAN with documentation available at https://modeloriented.github.io/survex.

## 1 Introduction

Survival analysis focuses on the estimation of time-to-event distributions while considering the effects of censoring. Over time, this field has witnessed substantial progress, initially driven by conventional statistical approaches like the Cox proportional hazards model (Cox 1972). However, inherent limitations and challenges within this domain have spurred the integration of machine learning methodologies, introducing enhanced performance and flexibility (Wang *et al.* 2019). This convergence of statistics and machine learning is particularly evident in the R programming language (R Core Team 2022), where various frameworks have been developed to facilitate the application of both traditional statistical models and modern machine learning techniques for survival analysis tasks.

Despite the promising potential of integrating machine learning into biomedical research and healthcare, the opaque nature of black-box models has raised valid concerns (Ahmad *et al.* 2018). In response, interpretable machine learning and explainable artificial intelligence methods has emerged as a viable solution (Biecek and Burzykowski 2021, Molnar 2022), including software packages in R (Biecek 2018, Molnar *et al.* 2018). However, these software packages cannot handle censoring and do not provide explanations for survival models. To fill this gap, we propose ‘survex’ as an innovative solution that provides comprehensive explanations for entire models and individual predictions, alongside performance measures and a unified prediction interface. Operating within the R environment, ‘survex’ supports numerous packages with survival models while maintaining flexibility to integrate others. By integrating explanations into the modeling and analysis process, ‘survex’ aspires to empower stakeholders, particularly in critical domains like healthcare, with a deeper understanding of the model’s predictions and underlying rationales, ultimately promoting trust and informed decision-making.

## 2 Related work

In the domain of survival analysis, various software packages offer a range of statistical and machine learning methods. In R, an essential component is the ‘survival’ package (Therneau 2023), which contains the fundamental statistical models like the Cox proportional hazards (Cox 1972) and accelerated failure time (Kalbfleisch and Prentice 2002) models. Alongside this, ‘randomForestSRC’ (Ishwaran and Kogalur 2007) and ‘ranger’ (Wright and Ziegler 2017) packages provide implementations of the notable random survival forest algorithm (Ishwaran *et al.* 2008). Complementing these, the ‘survivalmodels’ package (Sonabend 2022) offers deep neural networks within the survival analysis paradigm. To streamline consistency within this diverse toolkit, frameworks such as ‘mlr3proba’ (Sonabend *et al.* 2021) and ‘censored’ (Hvitfeldt and Frick 2023) extend the ‘mlr3’ (Lang *et al.* 2019) and ‘parsnip’ (Kuhn and Vaughan 2023) frameworks, respectively, to provide standardized ways of using different survival models.

To illustrate the wide range of model explainability tools available in the R environment, it is pertinent to highlight packages such as ‘DALEX’ (Biecek 2018) and ‘iml’ (Molnar *et al.* 2018), which offer a diverse spectrum of XAI techniques. While there are many packages in this field, their core focus remains rooted in the domain of explaining classification and regression models. Adapting certain explanatory methods for survival models can be accomplished, but it requires careful adjustment due to the unique predictive nature of these models. To address this challenge, ‘survex’ offers specifically tailored explanations that incorporate the time dimension inherent in the survival models’ predictions. Furthermore, methods dedicated to explain survival models have been formulated, such as SurvLIME (Kovalev *et al.* 2020) with a Python implementation (Pachón-García *et al.* 2024) or SurvSHAP(t) (Krzyziński *et al.* 2023). The ‘survex’ package is equipped to incorporate also these advanced techniques, further enhancing its functionalities range. It is worth acknowledging the inspiration drawn from the ‘survxai’ package (Grudziaz *et al.* 2018), which has significantly influenced the development of ‘survex’. However, ‘survex’ offers a broader spectrum of functionalities, recently proposed explanation methods, and supports a wider range of models. In addition, ‘survxai’ is no longer maintained.

## 3 Implementation and functionalities

The following section presents a brief overview of the functionalities of ‘survex’. More details about the explanation methods and implementation can be found in the accompanying Supplementary data and package documentation.

**Figure 1.**
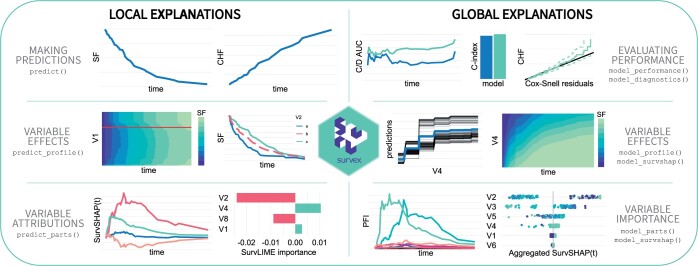
Explanations and functionalities available in the ‘survex’ package. The methods are divided into local (concerning individual predictions) and global (concerning the model). The diagram illustrates simplified examples of the visualizations of selected explanations in each category. A complete list of functionalities with documentation is available at https://modeloriented.github.io/survex.

The design of ‘survex’ draws inspiration from the ‘DALEX’ R package (Biecek 2018), serving as an extension tailored to explain survival models. This is exemplified in a methodically structured interface, aligned with the structure proposed by Baniecki *et al.* (2023a). The package facilitates the interpretation of survival models through its diverse functionalities, categorized into local and global contexts, as depicted in Fig. 1. ‘survex’ is engineered as a model-agnostic framework adaptable to any model that returns predictions in the form of a survival function or cumulative hazards function.

The model-agnostic approach is implemented by the central component of the package—the explainer object. Serving as a wrapper for survival models, it unifies their prediction interfaces and stores essential background data necessary for obtaining predictions and explanations. For models from widely-used packages, the explainer can be created automatically, by providing the model object to the explain() function. However, any model not adapted automatically by ‘survex’ can be explained by specifying the way of predicting the survival function. For the wrapped model, the unified prediction interface can be used by the predict() function, capable of generating predictions in the form of survival function, cumulative hazard function, or relative risk.

### 3.1 Explanation methods

Global explanations, concerning the whole model (dataset level), are marked with the model prefix, while local explanations, referring to individual predictions (observation level), are denoted by the predict prefix. Computing an explanation involves invoking the relevant function with the explainer object as the primary argument, supplemented by additional details. One of the key parameters is the output_type—the default selection of ‘survival’ generates time-dependent explanations based on a survival function. However, it is also possible to select ‘chf’ for explanations related to cumulative hazard function or ‘risk’, which results in more standard explanations based on a prediction in the form of relative risk (single number).

The model_parts() function outputs variable importance scores for the model. It leverages permutation variable importance (Breiman 2001, Fisher *et al.* 2019), i.e. quantifies the extent by which performance metric values are impacted upon permuting values of a chosen predictor. Performance measures can also be used with the model_performance() function, allowing users to comprehensively evaluate the models’ predictive capabilities. This function also offers the possibility to prepare Receiver Operating Characteristic (ROC) curves at different time points, by treating the survival probability at a selected time point as the response for the classification task.

Furthermore, the model_diagnostics() function facilitates diagnostic assessments through analysis of the residuals. It supports the calculation and visualization of martingale residuals, deviance residuals (Therneau *et al.* 1990), and Cox–Snell residuals (Cox and Snell 1968).

Explanations obtained by the model_profile() function reveal the influence of a specific variable on the model’s predictions. They are constructed based on one of two distinct methodologies: partial dependence plots (Friedman 2001) or accumulated local effects (Apley and Zhu 2020). Moreover, for insight into potential interaction effects, ‘survex’ offers the model_profile_2d() function, which generates profiles for two variables.

Using the predict_parts() function results in explanations that reveal the contributions of variables to a model’s prediction for a selected observation. In ‘survex’, these insights can be obtained using one of the two methodologies. The default method is SurvSHAP(t) (Krzyziński *et al.* 2023), leveraging SHAP values applied to the survival function to give the variable attributions at different times, alongside their aggregations over time. Alternatively, the SurvLIME approach fits a surrogate Cox model in the local neighborhood of the selected observation and uses its coefficients as the explanation.

The predict_profile() function is related to explanations concerning a single variable’s impact on a specific prediction. These insights are derived via the individual conditional expectation method (Goldstein *et al.* 2015), also known as the *ceteris paribus* method, as it involves altering the values of a single variable while keeping all others constant. These results can be analyzed together with partial dependence plots, which are their average.

The model_survshap() function streamlines the process of accessing SurvSHAP(t) explanations for a specified set of observations. Beyond individual explanations, this function aggregates SurvSHAP(t) values, revealing global insights into the model’s behavior. Moreover, it incorporates accessible visualization methods, including SurvSHAP(t) bee swarm and dependence plots, inspired by the well-established ‘shap’ Python package (Lundberg and Lee 2017).

It should be noted that methods using permute-and-predict mechanism have been criticized for producing misleading results when dealing with strongly correlated variables (Hooker *et al.* 2019), and there are alternative methods specifically designed to address these challenges (Delicado and Peña 2019). Thus, techniques like permutation variable importance, individual conditional expectation and partial dependence plots should be used cautiously.

### 3.2 Visualizations

Within ‘survex’, various visualizations accompany its explanations. Plots are prepared using the ‘ggplot2’ package (Wickham 2016) through the implemented plot() function called on the object returned by the explanation. Extensive user customization is enabled by adjustable parameters within the plotting functions, augmented with advanced functions available in ‘ggplot2’. Plots can be created for multiple explainer objects at once, allowing the user to compare and differentiate the explanations for different models and observations.

### 3.3 Applications

‘survex’ has already demonstrated its applicability in the field of biomedical research and healthcare. Chen *et al.* (2023) employed the package to find out the relative importance of variables in survival models predicting sporadic pancreatic cancer. Nachit *et al.* (2023) used it to analyze partial dependence plots of different body composition parameters extracted from computer tomography scans in a random survival forest. Additionally, we successfully applied ‘survex’ to explain model bias in predicting hospital length of stay (Baniecki *et al.* 2023b).

## 4 Future work

Currently, ‘survex’ handles the most common case of right-censored data with a single type of event. However, a future roadmap envisions its extension to cover alternative censoring types and accommodate competing risk models. Moreover, ‘survex’ can be easily extended with additional explanation techniques, e.g. counterfactual explanations proposed by Kovalev *et al.* (2021), or more adaptations of existing methods known from classification and regression tasks.

## Supplementary Material

btad723_Supplementary_Data
