# Supplementary material for: survex: an R package for explaining machine learning survival models
Source: Bioinformatics. 2023 Dec 1;39(12):btad723. doi: 10.1093/bioinformatics/btad723 (PMC11025379; doi:10.1093/bioinformatics/btad723)
Supplement: btad723_Supplementary_Data [file btad723_supplementary_data.pdf]

Supplementary Information for survex: an R package for explaining machine learning survival models

Mikołaj Spytek   Mateusz Krzyziński   Sophie Hanna Langbein   Hubert Baniecki
Marvin N. Wright   Przemysław Biecek

A Unifying survival models

The model-agnostic philosophy of **survex** is realized through the `explainer` object, which unifies different survival models. This wrapper can be created automatically using the `explain()` function for many models implemented in widely-used R packages like **survival** (Therneau, 2023), **flexsurv** (Jackson, 2016), **rms** (Harrell Jr, 2023), **ranger** (Wright and Ziegler, 2017), or **randomForestSRC** (Ishwaran and Kogalur, 2007), as well as models encapsulated in comprehensive frameworks **mlr3proba** (Sonabend et al., 2021) and **censored** (Hvitfeldt and Frick, 2023). Additionally, **survex** extends its capabilities to explain models originating from the popular **scikit-survival** Python package (Pölsterl, 2020) when utilized within R via **reticulate** (Ushey et al., 2023). Collectively, this extensive support enables the automatic creation of explainers for more than 30 distinct survival models. However, **survex** also allows for creating custom explainers using the `explain_survival()` function, which can be used to prepare wrappers for models that are not adapted automatically.

A fully functional explainer includes the model object, background data (with explanatory variables) used to create the explanation and the response vector. It can produce a prediction in three different forms: a survival function (SF), a cumulative hazard function (CHF), and a risk score. As not all models have this capability inherently, the mathematical relation between SF and CHF, i.e., $\mathrm{SF}(t) = \exp\{-\mathrm{CHF}(t)\}$, is used where necessary, while the risk score is calculated as a sum from the CHF values evaluated at selected time points.

Time points play a crucial role in time-dependent predictions and explanations. Users can specify them manually to tailoring the analysis to their specific needs. However, by default, **survex** calculates the vector of time points based on a Kaplan-Meier estimator (Kaplan and Meier, 1958) computed for the given response. Specifically, fifty uniformly distributed survival quantiles and an additional time point representing the median survival time (if feasible) are selected.

B Creating explanations

Wrapping the model into an `explainer` object is convenient for creating explanations. They are calculated after the relevant function is called with an explainer given as the first argument. **survex** follows the taxonomy that divides explanations into two main types: global and local. Local explanations are marked with the `predict` prefix and are described in Section C. Global explanations are denoted with the `model` prefix and presented in Section D. Suffixes in function names are based on a naming convention previously used in the **DALEX** package (Biecek, 2018) and described as the grammar of interactive explanatory model analysis by Baniecki et al. (2023).

In the subsequent sections C and D, we describe the intuition and mathematical background related to methods offered by **survex**. We focus on time-dependent explanation methods more specific for survival models, as the techniques working with single-number prediction models used in classification and regression have already been described (Biecek and Burzykowski, 2021; Molnar, 2022). We limit ourselves to basic implementation details as more detailed descriptions of functions and their arguments, along with code examples, are available in the package documentation. There are also vignettes showing step-by-step how

to use the tool for specific use cases. Documentation, which is continuously updated, is available at `https://modeloriented.github.io/survex/`.

In mathematical description, we adopt the following notation. We use $f_t(\mathbf{x})$ to denote a prediction for an observation $\mathbf{x} = [\mathbf{x}^1, \ldots, \mathbf{x}^p]$ at time point t. The function f is estimated by the model of interest, and t ranges from t_0 to t_k (the vector of time points contained in the explainer). Unless otherwise stated, f can represent both survival function and cumulative hazard function, as the formulation of the methods is correct in both cases. When a differentiation is required, we use $f_t^{\mathrm{SF}}(\mathbf{x})$ for the survival function and $f_t^{\mathrm{CHF}}(\mathbf{x})$ for the cumulative hazard function. Moreover, we denote by $\mathbf{X}$ the whole background dataset consisting of n observations $\mathbf{x}_1, \mathbf{x}_2, \ldots, \mathbf{x}_n$, each having p variables. Further, by $\mathbf{y} = [(y_1, \delta_1), (y_2, \delta_2), \ldots, (y_n, \delta_n)]$, we denote the response – survival target (pairs of observed times and event indicators) for this dataset.

C Local explanation methods

C.1 Variable attributions

Variable attribution methods serve to indicate how different variables contribute to the prediction obtained from the model for the selected observation. **survex** offers two different methods SurvSHAP(t) and SurvLIME, which can be accessed through the `predict_parts()` function with specified `type` argument.

SurvSHAP(t). The default `type` in the `predict_parts()` function is `'survshap'`, which refers to the SurvSHAP(t) method (Krzyziński et al., 2023). This technique is inspired by the SHAP method (Lundberg and Lee, 2017) and adopts a game theoretical approach to estimate the contribution of each variable to the prediction of the model for a selected observation. The method was the first, specifically proposed for explaining survival models that provides so-called *time-dependent* explainability. It does so via estimating variable attributions at every selected time point, i.e., for the selected variable j and time point t, the technique estimates $\phi_t(\mathbf{x}, j)$.

The attributions can be calculated using various algorithms, including the KernelSHAP algorithm available through the **kernelshap** package (Mayer and Watson, 2023) or as an internal implementation, and the TreeSHAP algorithm (Lundberg et al., 2020) utilizing the **treeshap** package (Komisarczyk et al., 2023).

The calculated attributions are accurate, which means that after incorporating the mean prediction $\phi_t^0 = \frac{1}{n}\sum_{i=1}^{n} f_t(\mathbf{x}_i)$, they sum up to the model's prediction, i.e.

$$f_t(\mathbf{x}) = \phi_t^0 + \sum_{i=1}^{p} \phi_t(\mathbf{x}, i). \tag{1}$$

Furthermore, for each variable, the resulting SurvSHAP(t) function can be aggregated to determine the local importance. One way to do it is to average absolute values over time by integrating, i.e.,

$$\psi(\mathbf{x}, j) = \int_{t_0}^{t_k} |\phi_t(\mathbf{x}, j)| \, \mathrm{d}w(t), \tag{2}$$

where $w(t)$ represents a weight function, by default $w(t) = \frac{t}{t_k}$.

SurvLIME. Another available `type` is `'survlime'`, which corresponds to the SurvLIME method (Kovalev et al., 2020), drawing inspiration from the LIME technique (Ribeiro et al., 2016). SurvLIME aims to explain the prediction of the black-box survival model by its local approximation using a simple, interpretable surrogate model. For this purpose, a Cox proportional hazards model (Cox, 1972) is used, and it operates on the set of synthetic observations $\mathbf{x}_1^s, \mathbf{x}_2^s, \ldots, \mathbf{x}_{n_s}^s$ generated randomly within the neighborhood of the observation to be explained (using Gaussian noise).

The variables' attributions are determined using the coefficients of the Cox model. The authors of the method proposed to use the raw coefficient vector $\psi(\mathbf{x}) = [\psi(\mathbf{x}, 1), \psi(\mathbf{x}, 2), \ldots, \psi(\mathbf{x}, p)]$, which is calculated in the following optimization problem:

$$\psi(\mathbf{x}) = \arg\min_{\mathbf{b}} \sum_{i=1}^{n} w_i \sum_{j=0}^{k} v_{i,j}^2 \left(\ln f_{t_j}^{\mathrm{CHF}}(\mathbf{x}_i) - \ln H_{t_j}^0 - \mathbf{b}^T \mathbf{x}_i \right)^2 (t_{j+1} - t_j), \tag{3}$$

where w_i are weights related to the distance between observation $\mathbf{x}_i^s$ and $\mathbf{x}$, while H^0 is the baseline cumulative hazard function estimated on the generated neighbourhood for the surrogate Cox model.

In **survex**, in order to make the attributions comparable between variables of different scales, the default option is to consider the coefficients multiplied by the values of the variables for the observation being explained, i.e.,

$$\tilde{\psi}(\mathbf{x}, j) = \psi(\mathbf{x}, j) \cdot \mathbf{x}^j. \tag{4}$$

Note that SurvLIME has a notable limitation related to the challenges associated with the notion of an observation's neighborhood. Both the generation of new observations and the calculation of their weights may pose difficulties. In the **survex** implementation, the default approach mirrors that of the **lime** Python package. It employs Gaussian sampling and an exponential kernel with a default width set at $b = 0.75\sqrt{p}$. There is also an alternative option, suggested by Pachón-García et al. and used in the **SurvLIMEpy** package. This alternative method draws inspiration from non-parametric density estimation theory and employs a kernel width of $b = (\frac{4}{n(p+2)})^{\frac{1}{p+4}}$.

C.2 Variable dependence

Local variable dependence methods aim to understand the relationship between different variables and the model's prediction for a selected observation, focusing on the effects of a change in the variable on the outcome. For this purpose, **survex** implements individual conditional expectation curves (ICE curves), also known as Ceteris Paribus profiles (CP profiles), in the `predict_profile()` function.

The `predict_profile()` function calculates ICE profiles for a specific observation as proposed by Goldstein et al. (2015). Minor adjustments to the fundamental method are requisite to incorporate the temporal dimension present in the survival setting. The ICE profile function describes the dependence of the (approximated) conditional expected value (prediction) of the outcome on some realization z of variable j, using the prediction function f at a specific time point t, that is

$$\mathrm{ICE}_t(f, \mathbf{x}, j, z) = f_t(\mathbf{x}^{j|=z}). \tag{5}$$

Note that, $\mathbf{x}^{j|=z}$ denotes the observation $\mathbf{x}$ with the realization of variable j set to value z, which assumes values over the entire observed range for the variable. In **survex**, by default 101 uniform grid points are sampled within the range of $[\min(\mathbf{x}^j), \max(\mathbf{x}^j)]$. All other explanatory variables retain their fixed values as specified by the observation of interest $\mathbf{x}$. Distinct ICE curves are obtained for different time points t.

D Global explanation methods

D.1 Measuring performance

survex enables to assess models' predictive capabilities using the `model_performance()` function, which calculates different metrics specific for survival models. By default, two different time-dependent metrics are calculated, along with their integrated versions and the widely used concordance index.

The first time-dependent metric is the Brier score (Brier et al., 1950; Graf et al., 1999), which for the time point t is calculated as

$$\mathrm{BS}_t(f, \mathbf{X}) = I(y_i > t)\frac{(1 - f_t^{\mathrm{SF}}(\mathbf{x}_i))^2}{\hat{G}(t)} + \frac{1}{n}\sum_{i=1}^{n} I(y_i \leqslant t \wedge \delta_i = 1)\frac{(0 - f_t^{\mathrm{SF}}(\mathbf{x}_i))^2}{\hat{G}(y_i)}, \tag{6}$$

where $1/\hat{G}(t)$ is the inverse probability of censoring weight estimated using Kaplan-Meier estimator. It can be understood as the mean squared error at the time point t with incorporated censoring information. The true value for the selected observation is 1 if the observed time is greater than t while it is equal to 0 if the observed time is lower or equal t and the event occurred. For this metric, lower scores are better.

Another implemented time-dependent metric is the cumulative/dynamic area under receiver operating characteristics curve (C/D AUC). At the time point t, it is calculated as

$$\mathrm{AUC}_t^{c/d}(f, \mathbf{X}) = \frac{\sum_{i=1}^{n}\sum_{j=1}^{n} I(y_j > t)I(y_i \leqslant t)\omega_i I(f_t^{\mathrm{SF}}(\mathbf{x}_j) \leqslant f_t^{\mathrm{SF}}(\mathbf{x}_i))}{(\sum_{i=1}^{n} I(y_i > t))(\sum_{i=1}^{n} I(y_i \leqslant t)\omega_i)}, \tag{7}$$

where $\omega_i = 1/\hat{G}(t)$. It measures how well the model of interest differentiate observations who experienced the event of interest by a time t (cumulative cases) from observations for which the event occurred after this time (dynamic controls). The higher the C/D AUC, the better.

Both time-dependent metrics can be also aggregated using integration over time from t_0 to t_k with a weight function $w(t)$ to obtain a single-number metric. By default $w(t) = \frac{t}{t_k}$. Another metric is the concordance index (C-index) (Harrell Jr et al., 1984), which is not time-dependent and utilizes the prediction in the form of risk score. It quantifies ranking of risk scores produced by the model of interest using the following formula:

$$\mathrm{C}(f, \mathbf{X}) = \frac{\sum_{i \neq j} I(f^{\mathrm{risk}}(\mathbf{x}_i) < f^{\mathrm{risk}}(\mathbf{x}_j))I(y_i > y_j)\delta_j}{\sum_{i \neq j} I(y_i > y_j)\delta_j}. \tag{8}$$

Note that only comparable pairs are considered and ties are not comparable. The higher values of C-index are better.

Additionally, the user can provide their own metric, which is facilitated by auxiliary functions enabling to adapt loss functions from **mlr3proba** (`loss_adapt_mlr3proba()`) and calculating an integral with respect to a selected weight function (`loss_integrate()`).

Moreover, the `model_performance()` function with `type` set to `'rocs'` makes it possible to create Receiver Operating Characteristic (ROC) curves for selected time points, by treating the values of the survival function at that time as probabilities of survival.

D.2 Diagnostics

The fit of the model of interest can also be evaluated by diagnostic analysis of its residuals. In **survex**, the `model_diagnostics()` function calculates multiple types of survival residuals, providing insights into the model's predictions and helping users diagnose any issues.

The first type of residuals computed are Cox-Snell residuals (Cox and Snell, 1968), which rely on predicted cumulative hazard functions. Specifically, for an observation $\mathbf{x}_i$, the corresponding Cox-Snell residual is defined as the cumulative hazard function's value at the observed time point y_i, i.e.,

$$r_i^C = f_{y_i}^{CHF}(\mathbf{x}_i). \tag{9}$$

These residuals have the property that if the model fits the data, residuals follow an exponential distribution with a rate parameter of $\lambda = 1$. Consequently, diagnostic analysis involves comparing the cumulative hazard estimator for residuals with that of the standard exponential distribution.

Cox-Snell residuals serve as the foundation for calculating the next type of residuals, known as martingale residuals (Therneau et al., 1990), which are defined as

$$r_i^M = \delta_i - r_i^C. \tag{10}$$

Positive values indicate that the event of interest occurred sooner than expected based on the model's prediction, while negative values imply the opposite.

Lastly, deviance residuals (Therneau et al., 1990) are estimated using martingale ones. Specifically, they are calculated as

$$r_i^D = \mathrm{sgn}(r_i^M)\sqrt{-2\{r_i^M + \delta_i \ln(\delta_i - r_i^M)\}}, \tag{11}$$

which makes them centered around 0 and, thus, easier to interpret.

Martingale and deviance residuals can be plotted against observed times, variable values, or each other. Analyzing these residuals may help detect outliers or functional dependencies on variables.

D.3 Variable importance

Variable importance methods allow to understand the time-dependent importance of individual variables for a survival model in a global context (not only for one prediction). Importance scores can be calculated in **survex** using the `model_parts()` function. It utilizes the permutation variable importance concept, i.e., describes the importance of j-th variable as a change in the loss function $\mathcal{L}$ caused by permutations of this variable in the dataset, i.e.,

$$\mathrm{PFI}_t(f, \mathbf{X}, j, \mathcal{L}, \mathbf{y}) = \frac{1}{B}\sum_{i=1}^{B}\left(\mathcal{L}(f, \mathbf{X}, \mathbf{y}) - \mathcal{L}(f, \mathbf{X}^{*j_i}, \mathbf{y})\right). \tag{12}$$

Here, $\mathcal{L}$ represents the loss function chosen to evaluate model performance, $\mathbf{X}^{*j_i}$ denotes the i-th permutation of variable j within the dataset $\mathbf{X}$, and B is the number of different permutations. Permuting a variable is supposed to simulate the loss of information associated with the variable.

By default, the Brier score is used as a loss function, but users can select other implemented metrics or provide their custom function to calculate the loss. If the chosen loss function returns a single value, as in the case of integrated metrics or C-index, then the variable importance scores are also not time-dependent.

Another option for the assessment of time-dependent variable importance is to use the global aggregated version of SurvSHAP(t). After calculating SurvSHAP(t) results for every considered observation, the absolute values obtained can be averaged. **survex** allows such a procedure through `model_survshap()` function. Furthermore, the results can be leveraged to analyze the distribution of individual variables' attributions concerning variable values, providing additional insights beyond importance scores alone.

D.4 Variable dependence

Global variable dependence methods provide a comprehensive view of how different variables affect the model's predictions for all observations. The **survex** package provides access to two distinct techniques – partial dependence plots and accumulated local effects plots – by utilizing the `model_profile()` and the `model_profile_2d()` functions with the appropriate type argument specification.

Partial Dependence. The default type in the `model_profile()` function is `'partial'`, which induces the computation of partial dependence (PD) profiles or plots with minor adjustments to the original method by Friedman (2001) for the inclusion of the time dimension present in the survival setting. The partial dependence function describes how the expected value of the model prediction varies with respect to a chosen explanatory variable, over a set of different, individually fixed grid points z, while all other variables

fluctuate following their respective marginal distributions. In practice, a one-dimensional PD profile is estimated by the mean of the ICE profiles for all n observations $\mathbf{x} \in \mathbf{X}$ from a dataset, that is

$$\mathrm{PDP}_t(f, \mathbf{X}, j, z) = \frac{1}{n} \sum_{\mathbf{x} \in \mathbf{X}} f_t(\mathbf{x}^{j|=z}). \tag{13}$$

Different PD profiles are obtained for different time points t. The `model_profile_2d()` generates two-dimensional profiles for two variables, by computing the average of ICE profiles obtained by varying two variables j and k over grid values within their respective observed range, while keeping all other variables fixed at their observed values for each specific observation.

Accumulated Local Effects. The second available type is `'accumulated'`, which refers to accumulated local (AL) profiles or effects, as introduced by Apley and Zhu (2020). Again minor adjustments are made to account for different temporal dimensions. Equivalent to PD profiles, AL profiles aim to quantify and visualize how changes in a specific variable affect model predictions over all observations; however, these two approaches diverge in their estimation methodologies. Instead of considering the average impact of a Ceteris Paribus change in a singular variable, AL profiles capture and accumulate the local effects of variations in that variable. This distinction is particularly significant in addressing the extrapolation issue inherent in PD profiles. It arises when predictions or inferences outside the range of the observed data are made, particularly in the presence of strongly correlated variables, potentially leading to unreliable variable effect estimates. The `model_profile()` function uses the estimation procedure proposed by Apley and Zhu (2020). Let $N_j(k) = \left(z^j_{k-1}, z^j_k\right]$ $(k = 1, \ldots, K)$ be a partition of the range of the observed values $\mathbf{x}^j$ of $\mathbf{X}$ into K intervals, with z^j_0 just below $\min(x^j_1, \ldots, x^j_N)$ and $z^j_K = \max(x^j_1, \ldots, x^j_N)$. Furthermore, $n_j(k)$ denotes the number of observations $\mathbf{x}^j_i$ falling into $N_j(k)$, with $\sum_{k=1}^K n_j(k) = n$ and $k_j(z)$ is the index of interval $N_j(k)$ in which z falls, then

$$\mathrm{ALE}_t(f, \mathbf{X}, j, z) = \sum_{k=1}^{k_j(z)} \frac{1}{n_j(k)} \sum_{i: x^j_i \in N_j(k)} \left\{ f_t(\mathbf{x}^{j|=z^j_k}) - f_t(\mathbf{x}^{j|=z^j_{k-1}}) \right\}. \tag{14}$$

Finite differences within ICE profiles, computed at the interval boundaries of distinct intervals $N_j(k)$ for a chosen observation, provide estimations of the local effects. These are then averaged across all observations where the observed value of $\mathbf{X}^j$ falls within the respective interval, and subsequently, they are cumulatively accumulated over preceding intervals. Note, that in **survex** by default AL profiles are not centered, thus enabling direct comparisons with PD profiles; however, centered plots can be obtained by setting the `'center'` argument to `TRUE`.

Supplementary references

D. W. Apley and J. Zhu. Visualizing the Effects of Predictor Variables in Black Box Supervised Learning Models. *Journal of the Royal Statistical Society Series B*, 82(4):1059–1086, 2020. doi: 10.1111/rssb.12377.

H. Baniecki, D. Parzych, and P. Biecek. The Grammar of Interactive Explanatory Model Analysis. *Data Mining and Knowledge Discovery*, pages 1–37, 2023.

P. Biecek. DALEX: Explainers for Complex Predictive Models in R. *Journal of Machine Learning Research*, 19(84):1–5, 2018.

P. Biecek and T. Burzykowski. *Explanatory Model Analysis*. CRC Press, 2021. URL https://ema.drwhy.ai/.

G. W. Brier et al. Verification of Forecasts Expressed in Terms of Probability. *Monthly Weather Review*, 78(1):1–3, 1950.

D. R. Cox. Regression Models and Life-Tables. *Journal of the Royal Statistical Society. Series B*, 34(2): 187–220, 1972.

D. R. Cox and E. J. Snell. A General Definition of Residuals. *Journal of the Royal Statistical Society: Series B*, 30(2):248–265, 1968. doi: h10.1111/j.2517-6161.1968.tb00724.x.

J. H. Friedman. Greedy Function Approximation: a Gradient Boosting Machine. *Annals of Statistics*, pages 1189–1232, 2001.

A. Goldstein, A. Kapelner, J. Bleich, and E. Pitkin. Peeking Inside the Black Box: Visualizing Statistical Learning With Plots of Individual Conditional Expectation. *Journal of Computational and Graphical Statistics*, 24(1):44–65, 2015. doi: 10.1080/10618600.2014.907095.

E. Graf, C. Schmoor, W. Sauerbrei, and M. Schumacher. Assessment and Comparison of Prognostic Classification Schemes for Survival Data. *Statistics in Medicine*, 18(17–18):2529–2545, 1999.

F. E. Harrell Jr. *rms: Regression Modeling Strategies*, 2023. URL `https://CRAN.R-project.org/package=rms`. R package version 6.7-1.

F. E. Harrell Jr, K. L. Lee, R. M. Califf, D. B. Pryor, and R. A. Rosati. Regression Modelling Strategies for Improved Prognostic Prediction. *Statistics in Medicine*, 3(2):143–152, 1984.

E. Hvitfeldt and H. Frick. *censored: 'parsnip' Engines for Survival Models*, 2023. URL `https://github.com/tidymodels/censored`. R package version 0.2.0.

H. Ishwaran and U. B. Kogalur. Random Survival Forests for R. *R News*, 7(2):25–31, 2007.

C. Jackson. flexsurv: A Platform for Parametric Survival Modeling in R. *Journal of Statistical Software*, 70(8):1–33, 2016.

E. L. Kaplan and P. Meier. Nonparametric Estimation from Incomplete Observations. *Journal of the American Statistical Association*, 53(282):457–481, 1958.

K. Komisarczyk, P. Kozminski, S. Maksymiuk, and P. Biecek. *treeshap: Compute SHAP Values for Your Tree-Based Models Using the 'TreeSHAP' Algorithm*, 2023. URL `https://CRAN.R-project.org/package=treeshap`. R package version 0.3.0.

M. S. Kovalev, L. V. Utkin, and E. M. Kasimov. SurvLIME: A Method for Explaining Machine Learning Survival Models. *Knowledge-Based Systems*, 203:106164, 2020.

M. Krzyziński, M. Spytek, H. Baniecki, and P. Biecek. SurvSHAP(t): Time-dependent Explanations of Machine Learning Survival Models. *Knowledge-Based Systems*, 262:110234, 2023.

S. M. Lundberg and S.-I. Lee. A Unified Approach to Interpreting Model Predictions. In *Advances in Neural Information Processing Systems*, pages 4765–4774, 2017.

S. M. Lundberg, G. Erion, H. Chen, A. DeGrave, J. M. Prutkin, B. Nair, R. Katz, J. Himmelfarb, N. Bansal, and S.-I. Lee. From Local Explanations to Global Understanding with Explainable AI for Trees. *Nature Machine Intelligence*, 2(1):2522–5839, 2020.

M. Mayer and D. Watson. *kernelshap: Kernel SHAP*, 2023. URL `https://CRAN.R-project.org/package=kernelshap`. R package version 0.3.8.

C. Molnar. *Interpretable Machine Learning*, 2nd edition, 2022.

C. Pachón-García, C. Hernández-Pérez, P. Delicado, and V. Vilaplana. SurvLIMEpy: A Python Package Implementing SurvLIME. *Expert Systems with Applications*, 237:121620, 2024.

S. Pölsterl. scikit-survival: A Library for Time-to-Event Analysis Built on Top of scikit-learn. *Journal of Machine Learning Research*, 21(212):1–6, 2020.

M. T. Ribeiro, S. Singh, and C. Guestrin. "Why Should I Trust You?": Explaining the Predictions of Any Classifier. In *Proceedings of the 22nd ACM SIGKDD International Conference on Knowledge Discovery and Data Mining, San Francisco, CA, USA, August 13-17, 2016*, pages 1135–1144. ACM, 2016.

R. Sonabend, F. J. Király, A. Bender, B. Bischl, and M. Lang. mlr3proba: An R Package for Machine Learning in Survival Analysis. *Bioinformatics*, 37(17):2789–2791, 2021.

T. M. Therneau. *A Package for Survival Analysis in R*, 2023. URL https://CRAN.R-project.org/package=survival. R package version 3.5-3.

T. M. Therneau, P. M. Grambsch, and T. R. Fleming. Martingale-based Residuals for Survival Models. *Biometrika*, 77(1):147–160, 1990. doi: 10.1093/biomet/77.1.147.

K. Ushey, J. Allaire, and Y. Tang. *reticulate: Interface to 'Python'*, 2023. URL https://CRAN.R-project.org/package=reticulate. R package version 1.34.0.

M. N. Wright and A. Ziegler. ranger: A Fast Implementation of Random Forests for High Dimensional Data in C++ and R. *Journal of Statistical Software*, 77(1):1–17, 2017.
